# Supplementary material for: Comparison of angle-closure detection between automated gonioscopy and anterior-segment optical coherence tomography
Source: PLoS One. 2025 Sep 15;20(9):e0332590. doi: 10.1371/journal.pone.0332590 (PMC12435641; doi:10.1371/journal.pone.0332590)
Supplement: Attachment — Submitted filename: Comments2025-5-20.docx [file pone.0332590.s002.docx]

Dear Authors,

I appreciate the authors’ effort to evaluate concordance between AS-OCT and GS-1 gonioscopic photography for angle-closure assessment. However, I have identified several critical methodological limitations that, in the present design and with available data, cannot be adequately addressed:

1. **Sample size and power calculation**
   You acknowledge in the Limitations section that the study’s sample size is small. However, no formal sample size or power calculation is reported. Without evidence that your study was sufficiently powered to detect clinically meaningful differences in concordance, the validity of your findings remains uncertain.
2. **Choice of comparison standard**
   Gonioscopy is rightly considered the gold standard for assessing angle closure. Yet your study compares AS-OCT and GS-1 images to one another, rather than directly to gonioscopic grading. Logically, to evaluate whether GS-1 photography can serve as a less invasive alternative, its results should be compared against gonioscopic examination, not AS-OCT.
3. **Inconsistent lighting conditions**
   AS-OCT examinations were performed under dark-room conditions, whereas GS-1 photographs were acquired in ambient light. These differing illumination environments can substantially affect angle appearance, rendering direct comparison inappropriate.
4. **Intra-examiner reliability during manual correction**
   During the AS-OCT post-processing stage, an examiner manually corrected the automated segmentation. It is unclear whether intra-examiner consistency of these corrections was assessed, and no reliability statistics are provided.
5. **Undefined reference standard in ROC analysis**
   In your ROC curve analysis, you do not specify which modality serves as the reference (gold standard). Without this clarification, the results cannot be interpreted.

Given these fundamental methodological flaws, we believe the manuscript is not suitable for further consideration in its present form. We encourage you to address these issues—particularly defining an appropriate gold standard, standardizing imaging conditions, and performing rigorous reliability and power analyses—before resubmitting, either to this journal or elsewhere.
