# Supplementary material for: Comparison of angle-closure detection between automated gonioscopy and anterior-segment optical coherence tomography
Source: PLoS One. 2025 Sep 15;20(9):e0332590. doi: 10.1371/journal.pone.0332590 (PMC12435641; doi:10.1371/journal.pone.0332590)
Supplement: Attachment — Submitted filename: Response to Reviewers.docx [file pone.0332590.s004.docx]

July 11, 2025

Dr. Jiro Kogo

Academic Editor

*PLOS ONE*

Revised manuscript: PONE-D-25-17859

Title: “Comparison of angle-closure detection between automated gonioscopy and anterior-segment optical coherence tomography”

Dear Dr. Kogo:

We are pleased to correspond with you again regarding the revision of our aforementioned manuscript, which has benefited from a review that has contributed to improving its academic value.

We are grateful to the reviewers and to you for the thorough review of our revised manuscript. We also appreciate the insightful comments provided by the reviewers, which have significantly helped to improve the quality of our manuscript.

We have revised the manuscript in accordance with both the specific and general requests, with the modified text highlighted in yellow in the revised manuscript. Below, please find our point-by-point responses to the reviewers’ comments and concerns. Our responses are provided in blue text.

RESPONSE TO REVIEWER’S COMMENTS

REVIEWER EVALUATION

We would like to thank the reviewers for their time and efforts in reviewing our manuscript and for providing comments that have considerably helped us to improve our manuscript.

Reviewer #1: I appreciate the authors’ effort to evaluate concordance between AS-OCT and GS-1 gonioscopic photography for angle-closure assessment. However, I have identified several critical methodological limitations that, in the present design and with available data, cannot be adequately addressed:

1. Sample size and power calculation

You acknowledge in the Limitations section that the study’s sample size is small. However, no formal sample size or power calculation is reported. Without evidence that your study was sufficiently powered to detect clinically meaningful differences in concordance, the validity of your findings remains uncertain.

Thank you for your valuable comment. We agree that conducting an a priori power analysis and determining the required sample size are essential aspects in clinical research. In this study, we performed an a priori power analysis based on Cohen’s kappa by using G*Power version 3.1.9.7 (Faul et al., 2007) to calculate the necessary sample size. Specifically, assuming a medium effect size (κ = 0.4), an alpha level of 0.05, and a desired power of 0.80, the minimum required sample size was calculated to be 45 eyes. This information has been clearly stated in the Methods section, as follows:

*“*An a priori power analysis was conducted using GPower version 3.1.9.7 [24] to determine the required sample size for Cohen’s κ analysis. Assuming a medium effect size (κ = 0.4), an alpha level of 0.05, and a desired power of 0.80, the minimum required sample size was calculated as 45 eyes.” (Page 10, lines 158-161)

24.	Faul F, Erdfelder E, Lang AG, Buchner A. G *Power 3: a flexible statistical power analysis program for the social, behavioral, and biomedical sciences. Behav Res Meth 2007;39:175-91. doi: 10.3758/bf03193146.

2. Choice of comparison standard

Gonioscopy is rightly considered the gold standard for assessing angle closure. Yet your study compares AS-OCT and GS-1 images to one another, rather than directly to gonioscopic grading. Logically, to evaluate whether GS-1 photography can serve as a less invasive alternative, its results should be compared against gonioscopic examination, not AS-OCT.

Thank you for your comment. We fully understand that gonioscopy is the gold standard to diagnose angle closure, and that a comparison of GS-1 results with gonioscopic examination is essential to validate the accuracy of the GS-1. However, in clinical practice, reports have indicated that gonioscopy is often underutilized owing to time and technical constraints. Therefore, alternative or complementary methods that can be performed by examiners other than physicians, such as orthoptists, are needed to screen for angle closure.

AS-OCT has already been demonstrated in several previous studies to be useful for the diagnosis of angle closure. Moreover, AS-OCT can be performed by orthoptists, and with practical clinical implementation in mind, we prioritized a comparison between the GS-1 and AS-OCT in this study.

We also recognize the importance of directly comparing the GS-1 with manual gonioscopy, and we have clearly stated this limitation in the Discussion section, as follows:

“Additionally, comparisons with manual gonioscopy were not performed, leaving uncertainty about the extent to which the GS-1 captures images of the angle in a more open state compared with manual gonioscopy. Therefore, future studies are needed for comparisons between the GS-1 and manual gonioscopy.” (Page 19, lines 300-303)

I also added a statement in the Introduction to emphasize the need for alternative or complementary examinations to gonioscopy that can be performed by examiners such as orthoptists, as follows:

“Gonioscopy allows for angle assessment via color imaging and facilitates both static and dynamic examinations, rendering it useful for the understanding of angle structures [5]. However, owing to the complexity of the procedure and the need for proficiency, it is not always performed in practice [5,6]. Moreover, the evaluation of gonioscopic features reportedly varies depending on the degree of proficiency [7]. Furthermore, capturing and storing numerous detailed gonioscopic images of the entire angle is difficult. These factors reveal a need for relatively non-invasive devices that can replace or supplement gonioscopes and can be operated by examiners such as orthoptists.” (Page 4, lines 46-53)

3. Inconsistent lighting conditions

AS-OCT examinations were performed under dark-room conditions, whereas GS-1 photographs were acquired in ambient light. These differing illumination environments can substantially affect angle appearance, rendering direct comparison inappropriate.

Thank you for your insightful comments. We consider this a very important point. In principle, the standard approach for the diagnosis of angle closure is to perform examinations under dark-room conditions, as the anterior chamber angle reportedly appears wider under bright conditions. Therefore, GS-1 examinations should ideally also be performed in a dark room. However, as the GS-1 device itself emits light during imaging, we judged that the room conditions would not make a difference. Consequently, we performed the GS-1 examinations under normal, light conditions. We acknowledge that this might have significantly influenced the results of our study, and we have described this point in the Discussion section, as follows:

“During GS-1 imaging, the device emits light, causing light to enter the participant’s pupil and potentially induce pupil constriction. Angles reportedly appear wider under bright conditions than they do under dark conditions [25,26]. Therefore, GS-1-based examinations, which are performed under bright conditions, may not be useful for screening or diagnosis of angle closure.” (Page 16, lines 232-236)

As mentioned earlier, the objective of this study was to evaluate the diagnostic capability of GS-1 gonioscopy to detect angle closure, specifically as a modality that can be performed by orthoptists. Similarly, AS-OCT can be operated by orthoptists and its utility in the diagnosis of angle closure has already been reported. Therefore, considering the feasibility of examinations performed by orthoptists, we prioritized a comparison between GS-1 gonioscopy and AS-OCT in this study; clinical implementation was our foremost concern.

4. Intra-examiner reliability during manual correction

During the AS-OCT post-processing stage, an examiner manually corrected the automated segmentation. It is unclear whether intra-examiner consistency of these corrections was assessed, and no reliability statistics are provided.

Thank you for your helpful comments. We agree that intra-examiner reproducibility of AS-OCT is an important factor to ensure its reliability. In this study, approximately 30% of the cases (19 eyes) were randomly selected to assess intra-examiner reproducibility of AS-OCT evaluations. The analysis demonstrated high agreement, with a per-image Cohen’s kappa of 0.895 (P<0.001) and a per-case kappa of 1.000 (P<0.001), indicating excellent intra-examiner consistency in this study.
 We have added the following sentences to the Methods and Results sections:

“To evaluate the repeatability of the manual correction process in AS-OCT post-processing, approximately 30% of the total cases were randomly selected for intra-examiner reliability analysis. The same examiner performed a second round of segmentation corrections on a different day under masked conditions (the examiner was blinded to the first assessment results). Agreement between the two assessments was evaluated using Cohen’s κ statistics on both a per-image and per-case basis.” (Pages 9-10, lines 149-154)

“In total, 19 eyes were re-evaluated for intra-examiner reliability. The per-image analysis yielded a Cohen’s κ of 0.895 (P<0.001), and the per-case analysis revealed perfect agreement with a kappa of 1.000 (P<0.001). These results indicate high repeatability and consistency of the examiner’s manual corrections in this study.” (Page 14, lines 193-196)

5. Undefined reference standard in ROC analysis

In your ROC curve analysis, you do not specify which modality serves as the reference (gold standard). Without this clarification, the results cannot be interpreted.

Given these fundamental methodological flaws, we believe the manuscript is not suitable for further consideration in its present form. We encourage you to address these issues—particularly defining an appropriate gold standard, standardizing imaging conditions, and performing rigorous reliability and power analyses—before resubmitting, either to this journal or elsewhere.

Thank you for your comment. We apologize for not clearly stating that AS-OCT was used as the reference standard in the ROC analysis to evaluate concordance with GS-1 gonioscopy. In response to your suggestion, we have now clarified this fact in the Methods section.

“Receiver operating characteristic (ROC) analysis was performed, using AS-OCT as the reference standard and the GS-1 classification results as the test variables. The area under the ROC curve (AUC) and its 95% confidence interval were calculated.” (Page 9, lines 143-145)

Furthermore, upon recalculating and reviewing the analyses, we discovered that the ROC curves had been calculated using GS-1 gonioscopy as the reference standard instead of AS-OCT in some cases. We re-confirmed that AS-OCT is the correct reference standard, and we have repeated the ROC analyses. The corrected results have been updated in Tables 2 and 3 and the related text (page 12, lines 183-187).

**Table 2. Comparison of GS-1 and AS-OCT Assessment of Angle Closure.**

|  | **n** | **Angle closure determined via GS-1 (%)** | **Angle closure determined via AS-OCT (%)** | **κ (95% CI)** | **AUC (95%CI)** |
| --- | --- | --- | --- | --- | --- |
| **Per image** | 1660 | 46.1 | 57.0 | 0.173 (0.128, 0.218) | 0.589 (0.562, 0.617) |
| **Per eye** | 53 | 33.9 | 60.4 | 0.151 (-0.076, 0.378) | 0.584 (0.428, 0.740) |
| AS-OCT: anterior-segment optical coherence tomography, AUC: area under the receiver operating characteristic curve, CI: confidence interval, GS-1: GS-1 Gonioscope. | | | | | |

**Table 3. Comparison of GS-1 and AS-OCT Assessment of Angle Closure by Quadrant.**

|  | Angle closure determined via GS-1 (%) | Angle closure determined via AS-OCT (%) | κ (95% CI) | AUC (95% CI) | |
| --- | --- | --- | --- | --- | --- |
| Temporal | 60.0 | 53.75 | 0.036 (-0.060, 0.130) | 0.518 (0.462, 0.574) | |
| Nasal | 22.0 | 59.81 | 0.206 (0.140, 0.271) | 0.619 (0.565, 0.672) | |
| Superior | 46.06 | 53.7 | 0.260 (0.168, 0.352) | 0.631 (0.578, 0.685) | |
| Inferior | 56.35 | 62.35 | 0.203 (0.109, 0.297) | 0.605 (0.548, 0.661) | |
| AS-OCT: anterior-segment optical coherence tomography, AUC: area under the receiver operating characteristic curve, CI: confidence interval, GS-1: GS-1 Gonioscope. | | | | |  |

Reviewer #2: The paper conducted an agreement analysis of angle-closure assessments between GS-1 and AS-OCT. While the experiments and analysis are clearly presented, a few issues remain in the explanations, such as unclear comparison restrictions and limitations in the dataset. The following concerns are raised:

1.Definition of angle closure in AS-OCT and GS-1: In AS-OCT, angles classified as “narrow” or “closed” are considered as angle closure, while GS-1 defines angle closure as the obscuration of the posterior trabecular meshwork for more than half of the eye. Could you clarify how the terms “narrow” or “closed” are specifically defined in AS-OCT?

Thank you for your insightful comments. The criteria for angle closure are indeed a critical aspect of this study. Although the criteria to determine angle closure upon AS-OCT were described in the Methods section, the specific numerical thresholds were not included. We have now added these detailed values.

“To assess angle closure, we used the STAR Analyzer program installed on the device. This program automatically classifies the 32 directions of the anterior chamber angle as open, narrow, or closed based on the angle-opening distance (AOD) at 500 μm anterior to the scleral spur (AOD500) (Fig 2). Cutoff values for the AOD500 in each direction are predefined in the program (ranging from 0.087 to 0.198 mm). If the AOD500 value is equal to or greater than the cutoff, the angle is classified as open. If it is below the cutoff, the angle is classified as narrow, and if it is 0 mm, it is classified as closed.” (Page 7, lines 103-110)

2.Comparison between AS-OCT slices and GS-1 areas: The comparison between AS-OCT slices and GS-1 areas using directional pairs is presented. However, both the Cohen’s Kappa and AUC are relatively low. When using AOD 500 as a threshold, why is a narrow angle also considered as angle closure, given that AS-OCT was originally found to have higher sensitivity for assessing angle closure severity?

Thank you for your comment. In this study, the definition of angle closure via GS-1 gonioscopy, based on previous reports, was the inability to observe the posterior trabecular meshwork. However, this definition relies on qualitative assessment, and since indentation gonioscopy cannot be performed with the GS-1 device, discerning whether the angle is simply narrow or actually appositional (closed) from the images alone is challenging.

Given this background, we determined that GS-1 gonioscopy can only provide a binary classification into “open” and “non-open” (including both narrow and closed angles). Therefore, in this study, we also combined “narrow” and “closed” classifications made via AS-OCT as “angle closure present” to maintain consistency and fairness in the comparison.

However, as AS-OCT allows for more sensitive and quantitative evaluation, we recognize that if standardized evaluation methods for GS-1 become established in the future, separately analyzing “narrow” and “closed” angles would be meaning ful. We have added this point as a supplementary note in the Discussion section, as follows:

“In this study, angles classified as either “narrow” or “closed” via AS-OCT were collectively considered as closed angles in comparison with GS-1 results. This approach was taken to ensure consistency and fairness in the comparison, given that GS-1 provides only qualitative, static images under bright conditions without dynamic or indentation assessment, complicating the discernment of true appositional closure and a merely narrow angle. Although AS-OCT measurements are more sensitive and quantitative, no standardized grading system for GS-1 results has been established, and subjective judgments can easily influence the interpretation. Therefore, we adopted a binary classification of “open” versus “non-open” in this study. However, more detailed comparisons, in which “narrow” and “closed” categories are separated should be considered in future research.” (Page 19-20, lines 306-316)

3.Lighting conditions and the comparison with dark-room AS-OCT: As mentioned in the discussion, GS-1 sheds light during the examination, which could be considered a light-examination condition. It is commonly agreed that the anterior angle appears narrower under dark-room conditions. Given this assumption, is the comparison with dark-room AS-OCT results truly valid? Could this lighting difference be a factor influencing the disagreement between the two measurements?

Thank you for your important comments. We consider the different imaging conditions highly significant. Indeed, GS-1 gonioscopy was performed under bright conditions. In principle, the standard approach to diagnose angle closure is to conduct examinations under dark-room conditions, as the angle appears wider under bright conditions. Therefore, GS-1 examinations should ideally also be performed in a dark room. However, as the GS-1 device itself emits light during imaging, we could not standardize the conditions between the two methods. This discrepancy likely influenced the results of our study, and we have described this point in the Discussion section, as follows:

“During GS-1 imaging, the device emits light, causing light to enter the participant’s pupil and potentially induce pupil constriction. Angles reportedly appear wider under bright conditions than they do under dark conditions [25,26]. Therefore, GS-1-based examinations, which are performed under bright conditions, may not be useful for screening or diagnosis of angle closure.” (Page 16, lines 232-236)

However, the primary objective of this study was to evaluate how effectively GS-1, which can be used by orthoptists, can be used to detect angle closure compared with AS-OCT, which has already been demonstrated to have high sensitivity. Both modalities can be performed by orthoptists, and we believe that their comparison has clinical significance as a practical consideration for real-world implementation. To emphasize this point, we have added the following sentences to the Introduction:

“Furthermore, capturing and storing numerous detailed gonioscopic images of the entire angle is difficult. Therefore, there is a need for relatively non-invasive devices that can replace or supplement gonioscopy and can be operated by examiners such as orthoptists.” (Page 4, lines 51-53)

“In addition, we are not aware of reports on the diagnostic value of GS-1 images for angle closure.” (Page 6, lines 81~82)

4. Limitations in dataset: Although the statistical analysis has been conducted and the sample size exceeds the minimum required, the number of participants is relatively small, and the distribution of participants is unclear.

Thank you for your comment. We recognize that the small sample size is a limitation of this study, and we have described this point in the Discussion section, as follows:

“This study has several limitations. The number of cases was limited, and larger investigations are necessary.” (Page 19, lines 299-300)

Additionally, all cases in this study involved phakic eyes, and we have added each AS-OCT parameter for the study group to Table 1.

**Table 1. Demographic Information of Patients.**

| Characteristics | Total (n=53) |
| --- | --- |
| Age, years | 71.3 ± 9.3 |
| Sex, female, number (%) | 42 (79.2％) |
| Number of right eyes (%) | 28 (52.8%） |
| Best-corrected visual acuity (logMAR) | -0.053 ± 0.19 |
| Spherical equivalent (D) | 0.77 ± 2.07 |
| Angle opening distance _temporal_ (mm) | 0.16 ± 0.68 |
| Angle recess area _temporal_ (mm^2^) | 0.076 ± 0.30 |
| Trabecular iris space area _temporal_ (mm^2^) | 0.70 ± 0.29 |
| Trabecular iris angle (degree) | 14.44 ± 7.07 |
| Anterior chamber distance (mm) | 2.22 ± 0.18 |
| Lens vault (mm) | 0.76 ± 0.20 |
| Values are presented as means ± standard deviations, unless otherwise indicated. logMAR: logarithm of the minimum angle of resolution, D: diopter. | |

Reviewer #3: Thank you for submitting this interesting and clinically relevant study comparing automated gonioscopy (GS-1) with AS-OCT for angle-closure detection. The study design is appropriate, and the topic is important for glaucoma diagnostics.

However, I recommend addressing the following key points before the manuscript is suitable for publication:

Low agreement between methods

• The observed inter-modality agreement is minimal (κ ≈ 0.17). Please provide a more critical discussion of the clinical implications of this low concordance. Should clinicians rely more on one modality?

Thank you for your comment. We acknowledge that the low level of agreement observed in this study is an important result. As mentioned in the Discussion section, several factors might have contributed to the low concordance. This includes differences in the criteria used to determine angle closure between GS-1 gonioscopy and AS-OCT. We have described this point as follows:

“The criteria for angle closure used with gonioscopy (both manual and automated), in previous reports and in this study, were based on the visibility of the posterior trabecular meshwork. Anatomically, the visibility of the posterior trabecular meshwork and the contact between the scleral spur and the iris are considered relatively comparable criteria. In contrast, the AOD500 and the visibility of the posterior trabecular meshwork are presumed to be structurally different. This discrepancy likely explains the lower concordance observed in this study compared with that in previous reports [20,22].” (Pages 16-17, lines 246-252)

Additionally, based on the results of this study, GS-1 gonioscopy, which is performed under bright conditions, may not be suitable for screening or diagnosis of angle closure. Therefore, we have added the following sentence to the Discussion:

“Therefore, GS-1-based examinations, which are performed under bright conditions, may not be useful for screening or diagnosis of angle closure.” (Page 16, lines 235-236)

Furthermore, we have also added a statement in the Discussion to note the need to standardize the criteria for angle closure between AS-OCT and GS-1 gonioscopy and to re-evaluate their concordance.

“Further studies are necessary to standardize the criteria for angle closure for both methods.” (Page 16, lines 243-244)

Therefore, we believe that AS-OCT is superior for the screening of angle closure. Accordingly, we have stated the following in the Discussion section:

“As AS-OCT can be performed in a dark room, it may be more useful than the GS-1 for the diagnosis of angle closure.” (Page 21, lines 326-327)

Inclusion/exclusion criteria and clinical parameters

• Please clarify whether patients were included consecutively or selectively, and provide more detail on patient characteristics such as lens status (phakic/pseudophakic), presence of other eye diseases (e.g., plateau iris), and axial length.

In this study, we included patients who visited Chukyo Eye Clinic from 2020 to 2024, were suspected of having angle closure based on a Van Herick grade ≤2 upon slit-lamp microscopy, and underwent both AS-OCT (CASIA2 Advance; TOMEY, Nagoya, Japan) and GS-1 examinations. We excluded patients with a history of peripheral iridotomy, prior intraocular surgery, or ocular diseases other than cataracts. We have added this information to the Methods section, as follows:

“In this study, the exclusion criteria were a history of peripheral iridotomy, intraocular surgeries such as cataract extraction, or ocular diseases other than cataracts. “(Page 6, lines 92-93)

Additionally, we have included detailed clinical characteristics of the enrolled patients in Table 1. However, as the primary objective of this study was to compare the assessment of angle closure between AS-OCT and GS-1 gonioscopy, axial length measurements were not included as an enrolment criterion, and the axial length data were not available for most patients; therefore, this parameter is not reported in this study.

**Table 1. Demographic Information of Patients.**

| Characteristics | Total (n=53) |
| --- | --- |
| Age, years | 71.3 ± 9.3 |
| Sex, female, number (%) | 42 (79.2％) |
| Number of right eyes (%) | 28 (52.8%） |
| Best-corrected visual acuity (logMAR) | -0.053 ± 0.19 |
| Spherical equivalent (D) | 0.77 ± 2.07 |
| Angle opening distance _temporal_ (mm) | 0.16 ± 0.68 |
| Angle recess area _temporal_ (mm^2^) | 0.076 ± 0.30 |
| Trabecular iris space area _temporal_ (mm^2^) | 0.70 ± 0.29 |
| Trabecular iris angle (degree) | 14.44 ± 7.07 |
| Anterior chamber distance (mm) | 2.22 ± 0.18 |
| Lens vault (mm) | 0.76 ± 0.20 |
| Values are presented as means ± standard deviations, unless otherwise indicated. logMAR: logarithm of the minimum angle of resolution, D: diopter. | |

Observer variability

• The manuscript would benefit from reporting intra- and interobserver variability, and from explaining whether repeat assessments were performed or whether consensus procedures were used to resolve discrepancies.

Thank you for your comment. In this study, GS-1 images were independently evaluated by two glaucoma specialists (R.A. and Y.T.), and when their assessments did not agree, a final decision was made through consensus discussion. This consensus process has been described in the Methods section, as follows:

“The images were captured from 16 directions, divided into halves, and independently reviewed by two glaucoma specialists (R.A. and Y.T.). Areas in which the posterior trabecular meshwork was obscured in more than half of the image (Scheie classification grades III–IV) were judged as indicative of angle closure. For discrepancies between the two reviewers, consensus was reached through discussion.” (Page 8, line 124-129)

However, the primary objective of this study was to evaluate the concordance between GS-1 gonioscopy and AS-OCT; therefore, we did not conduct formal assessments of intra- or inter-observer reliability, nor did we perform repeat assessments. We recognize these aspects as important topics for future investigation, and we have stated this limitation in the Discussion, as follows:

“Another limitation of this study is that we did not formally evaluate intra- and inter-observer agreement in the interpretation of GS-1 images. Although all images were independently assessed by two glaucoma specialists and final decisions were made through consensus in cases of disagreement, we did not calculate numerical agreement rates or have the same examiner perform repeat evaluations. In future studies, the incorporation of reliability analyses, using statistics such as Cohen’s κ or the intraclass correlation coefficient, may enhance the validity and reproducibility of the evaluation methods.” (Page 20, lines 316-322)

Reproducibility

• It's unclear if repeat measurements or quality control thresholds were applied during imaging or analysis.

In this study, no images were retaken. As a quality control criterion, GS-1 images in which the angle structures could not be identified and AS-OCT images in which the scleral spur could not be identified were excluded from the analysis. We have added the following sentence to the Methods section:

“However, GS-1 images in which angle structures could not be identified and AS-OCT images in which the scleral spur could not be identified were excluded from the analysis.” (Pages 8-9, lines 134-136)

Minor points

• Address minor formatting issues (e.g., font size inconsistencies between lines 53–60; spacing issues in Table 1).

Thank you for your comment. These issues have been addressed.

• Typo in line 215: “mat” → “may”.

Thank you for pointing this out. We corrected the typographical error.

We sincerely thank the academic editor and reviewers for their thorough and insightful review, which has significantly improved the quality of our paper. We remain open to any further revision requests and look forward to hearing from you.

Sincerely,

Yuki Takagi, MD

Department of Ophthalmology

Japan Community Healthcare Organization Chukyo Hospital

1-1-10 Sanjo, Minami-ku, Nagoya-city, Aichi Prefecture 457-8510, Japan

Telephone: +81-52-691-7151

Fax: +81-52-692-5220

Email: ytakagi@sanjogroup.jp
