# Supplementary material for: Comparison of angle-closure detection between automated gonioscopy and anterior-segment optical coherence tomography
Source: PLoS One. 2025 Sep 15;20(9):e0332590. doi: 10.1371/journal.pone.0332590 (PMC12435641; doi:10.1371/journal.pone.0332590)
Supplement: Attachment — Submitted filename: Gs-1Resposeletter_-R1.docx [file pone.0332590.s005.docx]

September 1, 2025

Dr. Jiro Kogo

Academic Editor

*PLOS One*

Revised manuscript: PONE-D-25-17859R1

Title: “Comparison of angle-closure detection between automated gonioscopy and anterior-segment optical coherence tomography”

Dear Dr. Jiro Kogo:

It is a pleasure to correspond with you again regarding the revision of our aforementioned manuscript, which received a favorable review.

We are grateful to the reviewers and the editor for their thorough evaluation of our revised manuscript. We also appreciate the insightful comments provided by the reviewer, which have greatly improved the quality of the work.

We have revised the manuscript in accordance with the specific and general requests, with the modified text highlighted in yellow in the revised manuscript. Below, please find our point-by-point responses to the reviewers’ comments and concerns. Our responses are provided in blue text.

RESPONSE TO REVIEWER’S COMMENTS

REVIEWER EVALUATION

We would like to thank the reviewers for their time and effort in reviewing our manuscript and for providing comments that have considerably helped us improve it.

Reviewer #2: We appreciate the authors’ thorough responses to the previous reviews. However, a few minor concerns remain, and we would appreciate further clarification within the manuscript.

1. In the conclusion section, the authors state that AS-OCT may be more useful than the GS-1 because it can be performed in a dark room. However, traditional gonioscopy is also performed under dark-room conditions, and the GS-1 is introduced as “having the potential to complement conventional gonioscopy”. Could the authors further clarify the rational and intended clinical role of the GS-1 in this context aside from being non-contact (since it was not mentioned after introduction section)? Specifically, what is the motivation for selecting the GS-1 as the comparison method against AS-OCT, rather than manual gonioscopy, given that both AS-OCT and GS-1 differ in imaging principles and lighting conditions?

Thank you for your valuable comments. We fully understand that gonioscopy is the gold standard for diagnosing angle closure and that comparing GS-1 findings with gonioscopic examination is essential to validate the accuracy of the GS-1. However, in real-world clinical practice, reports indicate that gonioscopy is often underutilized because of time and technical constraints. Therefore, there is a need for alternative or complementary methods that can be performed by examiners other than physicians, such as orthoptists, to screen for angle closure. The primary aim of the present study was to compare AS-OCT and GS-1, both of which can be performed by orthoptists. The need for equipment that can be operated by orthoptists as a potential alternative to manual gonioscopy is described in the Introduction as follows:

“Gonioscopy enables angle assessment via color imaging and facilitates static and dynamic examinations, making it useful for evaluating angle structures [5]. However, owing to the complexity of the procedure and the required proficiency, it is not always performed in clinical practice [5,6]. Moreover, the evaluation of gonioscopic features reportedly varies depending on examiner expertise [7]. Furthermore, capturing and storing numerous detailed gonioscopic images of the entire angle is difficult. These factors highlight the need for relatively noninvasive devices that can replace or supplement gonioscopy and can be operated by examiners such as orthoptists.” (page 4, lines 47-54)

Furthermore, to clarify the study objective, we have revised and expanded the Introduction with the following sentence:

“Therefore, in this study, we examined the concordance between angle-closure assessments based on GS-1 and AS-OCT, both of which can be performed by orthoptists.” (page 6, lines 80-82)

Regarding the clinical role of the GS-1, based on our findings, we consider it unsuitable for screening for angle closure. However, because it captures 360° color images of the entire angle, we believe it is useful for evaluating features such as pigmentation. Accordingly, we have revised and expanded the Discussion section as follows:

“Therefore, although GS-1 examinations performed under bright conditions allow 360° color imaging that is useful for documenting features such as pigmentation, they may not be suitable for screening or diagnosing angle closure.” (page 16, lines 231-233)

“However, for evaluating findings such as angle pigmentation, the GS-1’s capability to capture and archive 360° color images of the entire angle may be useful.” (page 20, line 324 to page 21, line 326)

Finally, we apologize if our wording caused confusion; the GS-1 is not a non-contact device—it is a contact device.

2. Although being frequently questioned in recent years, manual gonioscopy is still considered as a golden standard for angle closure examination, please justify how AS-OCT results in this case replace manual gonioscopy and be considered as a relative ‘true value’ in the current comparison.

Thank you for your comments. As noted above, we likewise consider manual gonioscopy to be the current gold standard. However, given that the aim of the present study was to compare AS-OCT and GS-1—both of which can be performed by orthoptists—we focused our analyses on these two modalities. We treated AS-OCT as a relative “true value” because prior reports have shown good sensitivity relative to manual gonioscopy and because GS-1 imaging is performed under bright conditions, which may underestimate angle closure. Nevertheless, since AS-OCT is not the true gold standard, we have revised and expanded the Discussion to acknowledge this limitation with the following statement:

“Comparisons with manual gonioscopy were not performed, and uncertainty remains regarding the extent to which the GS-1 captures the angle in a more open configuration compared with manual gonioscopy. In addition, because AS-OCT was used as a relative reference standard, verification bias is possible. Therefore, future studies are needed that include three-way comparisons among GS-1, manual gonioscopy, and AS-OCT.” (page 19, lines 296-300)

3. The manuscript is generally well-written, though minor grammatical refinements could help with better understanding.

Thank you for your helpful comments. We have carefully revised the manuscript for clarity and grammar.

We sincerely thank the academic editor, editors, and reviewers for their thorough and insightful review, which has greatly improved the quality of our paper. We remain open to any further revision requests and look forward to your response.

Sincerely,

Yuki Takagi, MD

Department of Ophthalmology

Japan Community Healthcare Organization Chukyo Hospital

1-1-10 Sanjo Minami-ku Nagoya-city, Aichi Prefecture, Japan

Telephone: +81-52-691-7151

Fax: +81-52-692-5220

Email: ytakagi@sanjogroup.jp
